# Induction of hippocampal long-term potentiation increases the morphological dynamics of microglial processes and prolongs their contacts with dendritic spines

**DOI:** 10.1038/srep32422

**Published:** 2016-09-08

**Authors:** Thomas Pfeiffer, Elena Avignone, U. Valentin Nägerl

**Affiliations:** 1 Interdisciplinary Institute for Neuroscience, UMR 5297 CNRS, Bordeaux, France; 2 Université de Bordeaux, Bordeaux, France

## Abstract

Recently microglia, the resident immune cells of the brain, have been recognized as multi-tasking talents that are not only essential in the diseased brain, but also actively contribute to synaptic circuit remodeling during normal brain development. It is well established that microglia dynamically scan their environment and thereby establish transient physical contacts with neuronal synapses, which may allow them to sense and influence synaptic function. However, it is unknown whether and how the morphological dynamics of microglia and their physical interactions with synapses are affected by the induction of synaptic plasticity in the adult brain. To this end, we characterized the morphological dynamics of microglia and their interactions with synapses before and after the induction of synaptic plasticity (LTP) in the hippocampus by time-lapse two-photon imaging and electrophysiological recordings in acute brain slices. We demonstrate that during hippocampal LTP microglia alter their morphological dynamics by increasing the number of their processes and by prolonging their physical contacts with dendritic spines. These effects were absent in the presence of an NMDA receptor antagonist. Taken together, this altered behavior could reflect an active microglial involvement in circuit remodeling during activity-dependent synaptic plasticity in the healthy adult brain.

A number of studies over the last few years have indicated that microglia carry out a variety of important functions in the healthy brain, leading to a reappraisal of their role for normal brain physiology^1^. Microglia have highly dynamic finger-like processes that are continuously moving through the surrounding brain tissue^2^,^3^. During this scanning-like activity, which is modulated by neuronal activity^2^, microglial processes establish transient contacts with synapses. While lowering neuronal activity reduces the number of contacts with synaptic structures, conditions of cerebral ischemia prolong microglia-synapse interactions^4^.

Microglia have been shown to remove synapses (‘synaptic stripping’) in lesion and inflammation models^5^,^6^, and recent evidence indicates that they contribute to synapse pruning during normal brain development, possibly by way of their phagocytic activity^7^,^8^. Moreover, microglia appear to be able to influence excitatory synaptic transmission by the release of modulatory factors like ATP^9^.

Given these observations it stands to reason that microglia might also contribute to activity-dependent synaptic plasticity in the healthy adult brain^1^. Evidence in support of such a role has come from Cx3Cr1^−/−^ mice, where the elimination of the fractalkine receptor (Cx3Cr1) from microglia was shown to disrupt hippocampal LTP^10^. Moreover, it was recently shown that microglial processes are preferentially steered towards active neurons^11^,^12^ and that their outgrowth is promoted by the activation of neuronal NMDARs^13^,^14^, which are in turn strongly activated during LTP induction. However, an earlier study did not detect any changes in microglial motility in response to glutamate applications or after the induction of LTP, arguing against microglia being involved in synaptic plasticity^15^.

Given these incongruent reports, we set out to revisit this issue by directly visualizing the morphological interactions between microglial processes and dendritic spines during synaptic plasticity. To this end, we combined two-photon time-lapse imaging with extracellular field recordings in acute hippocampal brain slices obtained from transgenic mice, where microglia and neurons were labeled by two different fluorophores. We analyzed the morphological dynamics of microglia and their dynamic interactions with dendritic spines of CA1 pyramidal neurons before and after the induction of hippocampal LTP.

We observed that microglia increased the number of their processes and that the duration of microglia-spine contacts increased after LTP induction. By contrast, in the presence of the NMDAR antagonist APV these changes were suppressed.

Our study provides clear evidence for microglia to be able to sense and react to the induction of synaptic plasticity, supporting the notion of a microglial contribution to activity-dependent changes at the synapse in the healthy adult brain.

## Results

### Microglial morphological dynamics are altered after induction of hippocampal LTP

**Figure 1:**
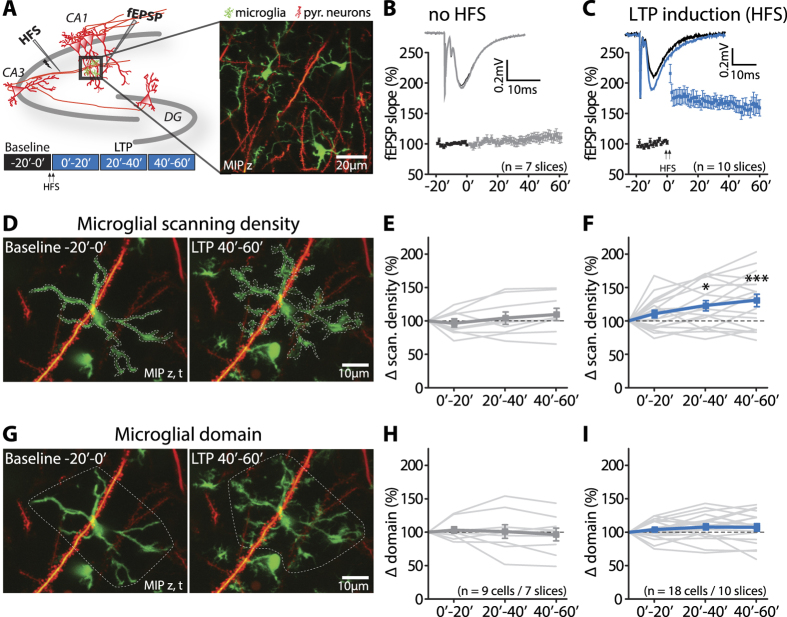
Microglial morphological dynamics are altered during hippocampal LTP. (**A**) Scheme of the experimental design, showing the location of the stimulation and recording electrodes and the region of interest for the concurrent image acquisitions in the *stratum radiatum* of hippocampal CA1. Representative region of interest with microglia (green) and pyramidal neurons (red). (**B**,**C)** Normalized fEPSP slope with (C, n = 10 slices) and without (B, n = 7 slices) the induction of LTP using a HFS (two arrows). The insets represent average fEPSPs in baseline (black), 40–60 min after LTP induction (blue) (**C**) and without application of a HFS (grey) (**B**). (**D**) Cumulative MIPs over 20 min show an increase in GFP-positive pixels, indicating an elevation in the scanning density 40–60 min after HFS (right) compared to baseline conditions (left). (**E**,**F**) The scanning density was constant over time in the absence of a HFS (**E**) (control: repeated measures Anova, factor of time: p = 0.3; n = 9 cells/7 slices), while it was significantly enhanced 20–40 min after LTP induction and remained elevated (40′–60′) (**F**) (LTP: repeated measures Anova and Turkey’s multiple comparisons post-test, factor of time: p = 0.0004; 0′–20′: p = 0.48; 20′–40′: p = 0.01; 40′–60′: p = 0.0004, compared to baseline; n = 18 cells/10 slices). (**G**) Cumulative MIPs showing that the microglial domain is unaffected 40–60 min after LTP induction (right) compared to baseline (left). (**H,I**) Microglial domain was stable and did not change over 80 min in absence (H) (control: repeated measures Anova, factor of time: p = 0.83; n = 9 cells/7 slices) and presence of the HFS (I) (LTP: repeated measures Anova, factor of time: p = 0.23; n = 18 cells/10 slices).

At first, we verified that time-lapse two-photon imaging together with recordings of evoked field potentials in *Stratum radiatum* of the CA1 area of the hippocampus was compatible with maintaining microglia in their ‘resting’ state (Fig. 1A,B). We observed microglia with stationary cell bodies and main branches giving rise to highly ramified and motile processes, which resembled those reported *in vivo*^2^,^3^. Prolonged time-lapse imaging (160 z-stacks acquired during 80 minutes) did not induce any signs of microglial activation or neuronal damage.

To investigate whether microglia can sense the induction of synaptic plasticity, we compared microglial morphological dynamics before and after the induction of LTP in hippocampal CA1, which was robustly expressed after electrical high-frequency stimulation (HFS) of the Schaffer collateral afferents (161.6% ± 1.1% of baseline, n = 10 slices, p < 0.001; Fig. 1C).

To globally quantify these dynamics, we counted the total number of GFP-positive pixels in maximum-intensity projections (MIP) of the two-photon image stacks across space and time (see Methods for details).

After application of the HFS, the number of GFP-positive pixels was significantly increased in the cumulative MIPs (Fig. 1D), which was not observed in control experiments without HFS over time (Fig. 1B,E) (number of GFP-positive pixels; control: Anova with repeated measures and Turkey’s multiple comparisons post-tests, factor of time: p = 0.3, 0′–20′: 97.1% ± 6.1%, p = 0.97; 20′–40′: 104.1% ± 9.2%, p = 0.93; 40′–60′: 109.6% ± 9.3%, p = 0.5, all compared to baseline; 0′–20′ vs. 20′–40′: p = 0.74, 0′–20′ vs. 40′–60′: p = 0.28, 20′–40′ vs. 40′–60′: p = 0.85; mean ± sem,. n = 9 cells/7 slices; Fig. 1E). This increase persisted for at least 60 minutes until the end of the recordings (LTP: Anova with repeated measures and Turkey’s multiple comparisons post-tests, factor of time: p = 0.0004, 0′–20′: 110.2% ± 5.9%, p = 0.48; 20′–40′: 122.7% ± 7.7%, p = 0.01; 40′–60′: 130.5% ± 9.4%, p = 0.0004, all compared to baseline; 0′–20′ vs. 20′–40′: p = 0.3, 0′–20′ vs. 40′–60′: p = 0.03, 20′–40′ vs. 40′–60′: p = 0.68; mean ± sem, n = 18 cells/10 slices; Fig. 1F).

By contrast, the size of microglial domains, as defined by the perimeter within which individual microglia project their dynamic processes, was unaffected by the induction of LTP (domain size; control: Anova with repeated measures and Turkey’s multiple comparisons post-tests, factor of time: p = 0.83, 0′–20′: 103.3% ± 4.9%, p = 0.97; 20′–40′: 101% ± 10.1%, p = 0.99; 40′–60′: 96.6% ± 9.6%, p = 0.96, all compared to baseline; 0′–20′ vs. 20′–40′: p = 0.99, 0′–20′ vs. 40′–60′: p = 0.79, 20′–40′ vs. 40′–60′: p = 0.93; n = 9 cells/7 slices; LTP: factor of time: p = 0.23, 0′–20′: 104.1% ± 3.5%, p = 0.76; 20′–40′: 108% ± 4.6%, p = 0.24; 40′–60′: 107.2% ± 5.7%, p = 0.32, all compared to baseline; 0′–20′ vs. 20′–40′: p = 0.79, 0′–20′ vs. 40′–60′: p = 0.88, 20′–40′ vs. 40′–60′: p = 0.99; mean ± sem, n = 18 cells/10 slices; Fig. 1G–I).

Taken together, we observed an increase in microglial morphological dynamics after the induction of LTP, as measured by the integrated number of GFP-positive pixels, while the perimeter within which individual microglia project their processes remained unchanged. In other words, the density at which microglia scanned the surrounding neuropil (‘scanning density’) was increased as the size of the scanned area (‘scanning domain’) stayed the same.

### LTP induction increases the number, but not the velocity, of microglial processes

**Figure 2:**
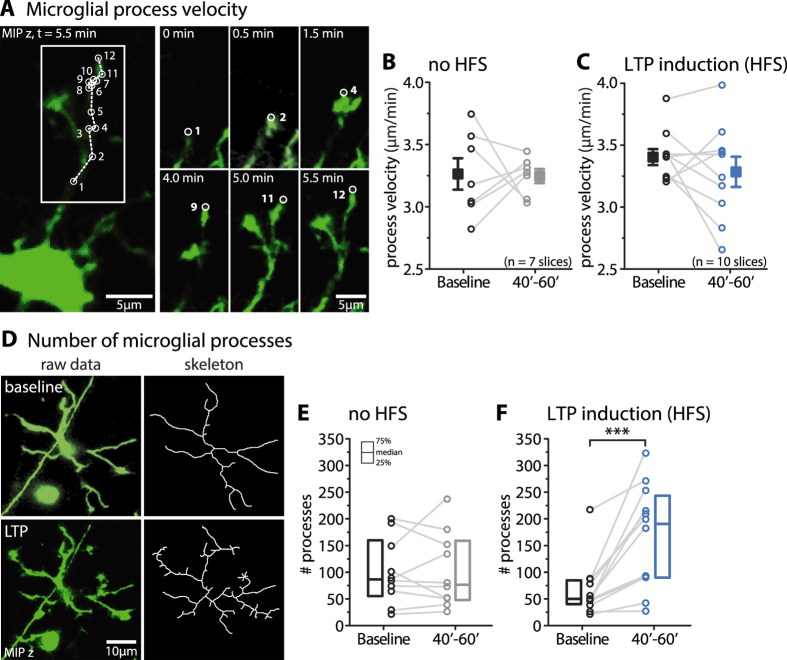
LTP induction increases the number, but not the velocity, of microglial processes. (**A**) Example of a tracked microglial process. The cumulative MIP is a projection over 12 consecutive time points, in which the microglial process tip was tracked over time (left panel, white circles). The magnified insets show the process tip at different time points (right panel). (**B,C**) The average velocity of microglial processes did not change over time in the absence of HFS (**B**) (baseline: 3.26 ± 0.13 μm/min; 40′–60′: 3.25 ± 0.06 μm/min; mean ± sem, paired t-test, p = 0.92, n = 7 slices) and after LTP induction (**C**) (baseline: 3.40 ± 0.07 μm/min; 40′–60′: 3.28 ± 0.12 μm/min; mean ± sem, paired t-test, p = 0.30, n = 10 slices). (**D)** MIP of a microglia in baseline and LTP conditions (left column) and the corresponding skeletons (right column). (**E**,**F**) Quantification of the number of processes per microglia in the absence of HFS (**E**) (baseline: median 86.5 [55.25, 159.75]; 40′–60′: median 76.5 [48.0, 159.0]; median [25^th^ and 75^th^ percentile], Wilcoxon paired test, p = 0.85, n = 10 cells/7 slices). Microglia increased the number of their processes upon LTP induction (baseline: median 50.0 [40.25, 84.75]; 40′–60′: median 190.5 [90.0, 243.5]; median [25^th^ and 75^th^ percentile], Wilcoxon paired test, p = 0.0004, n = 12 cells/9 slices).

The elevated scanning density by microglia could be due to an increase in the velocity or the number of individual microglial processes. To assess the relative contribution of these two factors we measured the velocity of single motile microglial processes (Fig. 2A) and counted the number of processes, during baseline and 40–60 min after the induction of LTP, which coincided with the largest increase in the microglial scanning density.

The average velocity did not change during the control experiments (i.e. in the absence of HFS) (baseline: 3.26 ± 0.13 μm/min; 40′–60′: 3.25 ± 0.06 μm/min; mean ± sem, paired t-test, p = 0.92, n = 7 slices; Fig. 2B). Likewise, we did not detect any change in microglial process velocity after LTP induction (baseline: 3.40 ± 0.07 μm/min; 40′–60′: 3.28 ± 0.12 μm/min; mean ± sem, paired t-test, p = 0.30, n = 10 slices; Fig. 2C).

We then examined whether microglia increased the number of their processes after LTP induction as increased microglial ramification was shown after pharmacological stimulation of neuronal NMDARs^13^. While the number of microglial processes remained constant during control conditions (baseline: median 86.5 [55.25, 159.75]; 40′–60′: median 76.5 [48.0, 159.0]; median [25^th^ and 75^th^ percentile], Wilcoxon paired test, p = 0.85, n = 10 cells/7 slices; Fig. 2E), it considerably increased after LTP induction (baseline: median 50.0 [40.25, 84.75]; 40′–60′: median 190.5 [90.0, 243.5]; median [25^th^ and 75^th^ percentile], Wilcoxon paired test, p = 0.0004, n = 12 cells/9 slices; Fig. 2D,F).

Taken together, we found that the increase in microglial morphological dynamics, or scanning density, after LTP induction can be accounted for by an increase in the number but not in the velocity of microglial processes.

### Microglia-spine interactions are altered during hippocampal LTP

**Figure 3:**
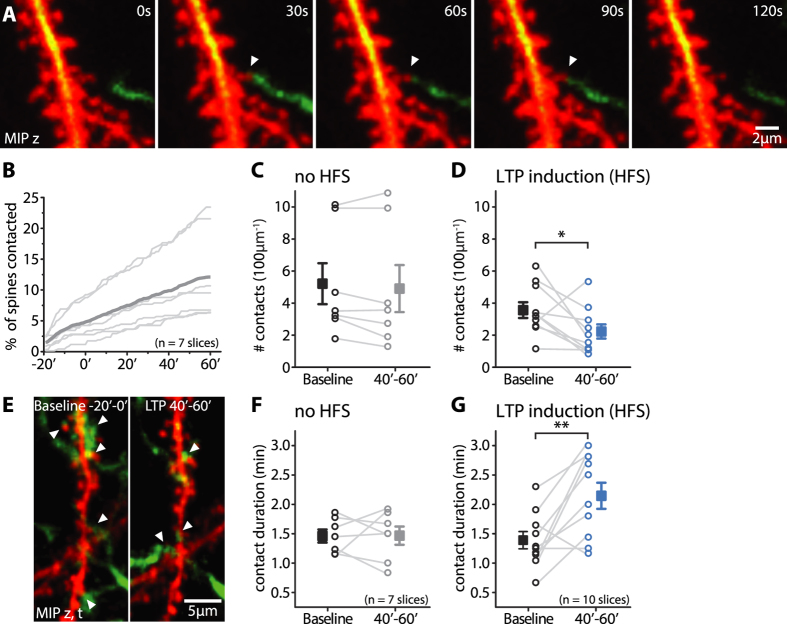
Microglia-spine interactions are modified after LTP induction. (**A**) A contact between a microglial process tip (green) and a dendritic spine (red) lasting for 90 s captured by time-lapse imaging (white arrow head, 2^nd^- 4^th^ image). (**B**) Percentage of spines contacted in the field of view during basal synaptic transmission, integrated over 80 min. (**C,D**) The number of contacts did not change over time in the absence of the HFS protocol (**C**) (# contacts per 100 μm dendrite observed during 20 min of baseline: 5.2 ± 1.2; during 40′–60′: 5.0 ± 1.4; mean ± sem, paired t-test, p = 0.25, n = 7 slices). The number of microglia-spine contacts was decreased 40–60 min after LTP induction (blue) compared to its baseline conditions (black) (**D**) (from 3.6 ± 0.4 to 2.2 ± 0.4 contacts; mean ± sem, paired t-test, p = 0.04, n = 10 slices). (**E**) MIPs over 20 min showing the number of microglia-spine contacts on a hippocampal dendrite in baseline (5 contacts) (left panel) and LTP conditions (3 contacts, 40′–60′) (right). (**F,G**) The duration of microglia-spine contacts was constant in the absence of a HFS protocol (**F**) (contact duration during baseline: 1.5 min ± 0.1 min; during 40′–60′: 1.5 min ± 0.2 min; mean ± sem, paired t-test, p = 0.96, n = 7 slices). In contrast, contact durations were significantly enhanced 40–60 min after the induction of LTP compared to baseline conditions (**G**) (from 1.4 min ± 0.1 min to 2.1 min ± 0.2 min; mean ± sem, paired t-test, p = 0.006, n = 10 slices).

While it was shown before that microglial processes can contact synapses^4^,^16^, little quantitative information exists on the frequency and dynamics of these interactions during basal synaptic transmission. To this end, we identified microglia-spine contacts, which we defined as a close physical apposition (i.e. <400 nm), in the time series of 3D image stacks, and analyzed their number and duration (Fig. 3A).

At the beginning of our experiments we observed only very few microglia-spine contacts: 1.61 ± 1.15 per 100 μm of dendrite (mean ± SD). Assuming a spine density of 1.1 spines per μm, which was reported for the *stratum radiatum* of the CA1 area^17^, this means that only 1.5% ± 1.0% (mean ± SD) of all spines are in contact with a microglial process at any given time (Fig. 3B). However, because of the high turnover of contacts, the cumulative percentage of contacted dendritic spines increased substantially, reaching on average 12.1% ± 7.3% after 80 min of time-lapse imaging, and in some slices even exceeded 20% (Fig. 3B).

Most of the detected contacts were transient during basal synaptic transmission and had short lifetimes (mean contact duration = 1.53 min ± 0.05 min; 390 contacts from 7 slices). Moreover, individual spines were rarely contacted twice within the observation period (4 out of 390 contacts). Although quite variable between different experiments, the average number of contacts was stable throughout the recording period (# contacts per 100 μm dendrite observed during 20 min of baseline: 5.2 ± 1.2; during 40′–60′: 5.0 ± 1.4; mean ± sem, paired t-test, p = 0.25, n = 7 slices; Fig. 3C). At the same time, the average duration of these contacts was stable throughout the experiments and similar across different experiments (contact duration during baseline: 1.5 min ± 0.1 min; during 40′–60′: 1.5 min ± 0.2 min; mean ± sem, paired t-test, p = 0.96, n = 7 slices; Fig. 3F).

Next, we assessed whether and how the induction of LTP affected the dynamics of microglia-spine contacts. We analyzed contacts in the time window 40–60 min after the induction of LTP, which coincided with the largest increase in microglial dynamics, and compared them to baseline before the HFS. We found that the number of contacts was reduced after HFS (from 3.6 ± 0.4 to 2.2 ± 0.4 contacts; mean ± sem, paired t-test, p = 0.04, n = 10 slices; Fig. 3D,E). By contrast, the duration of the formed contacts was on average increased by around 50% (from 1.4 min ± 0.1 min to 2.1 min ± 0.2 min; mean ± sem, paired t-test, p = 0.006, n = 10 slices; Fig. 3G). Thus, the induction of LTP led to fewer but more persistent microglia-spine contacts.

### HFS-induced effects depend on the activation of NMDA receptors

Subsequently, we tested whether the activity-dependent changes in the morphological dynamics of microglia and their interactions with dendritic spines depended on the activation of NMDA receptors. We repeated the experiments in the presence of the NMDAR antagonist APV, which blocks the expression of LTP at CA3/CA1 synapses, but does not appreciably affect pre- or postsynaptic firing behavior.

**Figure 4:**
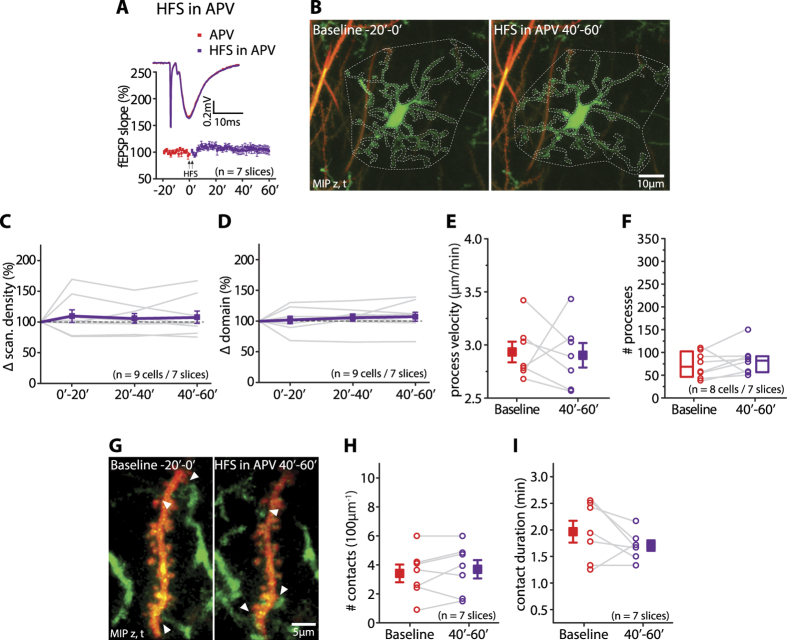
NMDAR antagonist APV prevents HFS-induced changes in microglial morphological dynamics and microglia-spine interactions. (**A**) Normalized fEPSP slope in the presence of APV during baseline (red) and after the HFS (purple), showing the suppression of LTP (n = 7 slices). The inset presents average fEPSPs traces in the presence of APV during the baseline (red) and 40–60 min after the HFS (purple). (**B**) Cumulative MIPs over 20 min in the presence of APV showing that the scanning density and domain 40–60 min after application of the HFS (right) were similar to its baseline conditions (left). (**C,D**) Scanning density (**C**) (repeated measures Anova, factor of time: p = 0.63; n = 9 cells/7 slices) and domain (**D**) (repeated measures Anova, factor of time: p = 0.5; n = 9 cells/7 slices) in the presence of APV did not change after the HFS. (**E,F**) In the presence of APV process velocity (**E**) (baseline: 2.93 ± 0.1 μm/min; 40′–60′: 2.9 ± 0.12 μm/min; mean ± sem, paired t-test, p = 0.83, n = 7 slices) and the number of processes (**F**) (baseline: median 68.5 [46.25, 102.25]; 40′–60′: median 82.0 [56.5, 91.5]; median [25^th^ and 75^th^ percentile], Wilcoxon paired test, p = 0.2, n = 8 cells/7 slices) remained unaffected by HFS. (**G**) Cumulative MIPs showing the number of microglia-spine contacts in the presence of APV during the baseline (3) and after the HFS (3, 40′–60′). (**H,I**) Application of the HFS in the presence of APV did not change the number of contacts (**H**) (baseline: 3.4 ± 0.6; during 40′–60′: 3.6 ± 0.6; mean ± sem, paired t-test, p = 0.32, n = 7 slices) nor their duration (**I**) (baseline: 2 min ± 0.2 min; during 40′–60′: 1.7 min ± 0.1 min; p = 0.16, n = 7 slices).

As expected, bath application of 50 μM APV completely blocked the expression of LTP (Fig. 4A). At the same time the HFS protocol did not induce any changes in microglial scanning density and scanning domain (scanning density: Anova with repeated measures and Turkey’s multiple comparisons post-tests, factor of time: p = 0.63, 0′–20′: 109.6% ± 10.1%; p = 0.6; 20′–40′: 106% ± 7.8%; p = 0.86; 40′–60′: 107.8% ± 10.1%, p = 0.74; all compared to baseline, 0′–20′ vs. 20′–40′: p = 0.96, 0′–20′ vs. 40′–60′: p = 0.99, 20′–40′ vs. 40′–60′: p = 0.99; n = 9 cells/7 slices; scanning domain: factor of time: p = 0.5, 0′–20′: 101.8% ± 6.1%; p = 0.99; 20′–40′: 105.7% ± 6%; p = 0.69; 40′–60′: 107.1% ± 7.2%; p = 0.53, all compared to baseline, 0′–20′ vs. 20′–40′: p = 0.87, 0′–20′ vs. 40′–60′: p = 0.74, 20′–40′ vs. 40′–60′: p = 0.99; mean ± sem, n = 9 cells/7 slices; Fig. 4B–D).

Likewise, in the presence of APV, the HFS did not induce any changes in microglial process velocity (baseline: 2.93 ± 0.1 μm/min; 40′–60′: 2.9 ± 0.12 μm/min; mean ± sem, paired t-test, p = 0.83, n = 7 slices; Fig. 4E) and in the number of microglial processes (baseline: median 68.5 [46.25, 102.25]; 40′–60′: median 82.0 [56.5, 91.5]; median [25^th^ and 75^th^ percentile], Wilcoxon paired test, p = 0.2, n = 8 cells/7 slices; Fig. 4F). At the same time, APV suppressed the changes in the number and duration of microglia-spine contacts that are triggered by the HFS protocol (contact number during baseline: 3.4 ± 0.6; during 40′–60′: 3.6 ± 0.6; mean ± sem, paired t-test, p = 0.32, n = 7 slices; Fig. 4G,H; contact duration during baseline: 2 min ± 0.2 min; during 40′–60′: 1.7 min ± 0.1 min; mean ± sem, paired t-test, p = 0.16, n = 7 slices; Fig. 4I).

In summary, the NMDAR antagonist APV inhibits the HFS-induced changes in microglial morphological dynamics and microglia-spine interactions.

## Discussion

We have shown that microglia establish transient contacts with a sizable fraction of dendritic spines within a relatively short period of time under baseline conditions in healthy adult brain tissue. Although the functional significance of these contacts is still elusive, we documented that the induction of synaptic plasticity altered these dynamic morphological interactions: while microglia exhibited enhanced morphological dynamics after LTP induction, they formed fewer but more stable contacts with dendritic spines. These changes suggest that microglia scan the surrounding neuropil more intensely by increasing the number of processes and that some of these processes engage more intimately with dendritic spines during synaptic plasticity.

We observed a gradual increase in microglial scanning density after induction of NMDAR-dependent LTP, which is consistent with reports showing that microglial motility is affected by changes in neuronal activity and NMDAR activation^2^,^13^,^14^. Given that microglia under normal conditions do not express functional NMDARs^14^, it is likely that activation of NMDARs on neurons due to the HFS and the concomitant increase in neuronal activity caused the changes in microglial morphological dynamics. In addition, the experiments indicate that the NMDAR antagonist APV did not have a direct effect on microglial morphology. The number of processes per microglia under baseline conditions was unaffected by the application of APV (see Supplementary Fig. S1, Figs 2E,F and 4F). The changes in microglial morphological dynamics after LTP induction became observable only 40 minutes after the HFS, and thus were more subtle and gradual than the rapid changes seen after glutamate and NMDA bath application^13^,^14^. We speculate that the activation of synaptic NMDARs during the synaptic plasticity paradigm is a more physiological stimulus compared with bath application of exogenous glutamate and NMDA, which may mimick pathological states.

Our findings contrast with an earlier study that failed to detect an effect of LTP induction on the morphology of microglia^15^. This discrepancy may be explained by important differences in experimental conditions. Unlike the previous confocal imaging study, which was conducted at room temperature, the temperature in our experiments was 33 °C, which is important to preserve normal morphological dynamics of microglia^18^. Moreover, our two-photon imaging approach in acute brain slices allowed us to image at depths beyond 50 μm, where microglial morphology is less affected by the slicing procedure^19^. Hence, we were able to image microglia continuously without interfering with their health state, as indicated by the control experiments where the ramifications of microglia stayed intact.

Microglial processes have been shown to associate with synaptic structures such as axonal boutons and dendritic spines in a neuronal activity-dependent way in the healthy adult brain^4^,^16^. Expanding these original observations, our study provides a quantitative description of the dynamics of these interactions sampled at a higher temporal resolution, which is needed to better assess the potential influence of microglia on synapses.

Microglia-spine contacts were rare and brief during basal synaptic transmission in hippocampal CA1. At any point in time, we estimated that only about 1.5% of CA1 hippocampal dendritic spines were in contact with a microglial process, which is consistent with a recent EM study^20^. While this percentage seems too low to be of physiological relevance, it must be kept in mind that EM analyses just offer a snap shot in time. As microglia were morphologically highly dynamic, over time they could effectively contact a substantial fraction of spines (>10% within 1.5 hours). In fact, they appeared to accomplish that in a swift and systematic way, as spines were usually contacted briefly (<1.5 min) and only once during the observation period (80 min). By extrapolation, this means that the vast majority of spines on CA1 pyramidal neurons might get visited by a microglial process over the course of a few hours.

Given the effect of LTP induction on microglial morphological dynamics, we wondered whether and how the dynamic interactions between microglial processes and dendritic spines might be affected. To our surprise, the number of microglia-spine contacts decreased after the induction of LTP. However, those contacts that did form became more stable, indicating that microglia engage more selectively and intimately with dendritic spines during LTP. More generally, these observations suggest that microglia can sustain at the same time increases in global dynamics and in contact stability with spines. Both phenomena may be independently regulated and could serve distinct purposes for the surrounding cellular network. Alternatively, the increase in the number of microglial processes might compensate for the more intense interactions with dendritic spines during neuronal plasticity, allowing microglia to maintain the level of surveillance activity.

What could be the role of microglia in synaptic plasticity? The change in contact duration during LTP is suggestive of an involvement of microglia in activity-dependent remodeling of synapses. During LTP, synapses undergo remodeling, which includes morphological and functional changes. Microglia could contribute to both phenomena. Conceivably, microglia utilize their phagocytic capacity to clear the extracellular space to accommodate growing spines and/or release modulatory factors that affect potentiated synapses. Conversely, microglial processes may also target synapses undergoing hetero-synaptic depression, which is a by-product of LTP, and contribute to the weakening or removal of these synapses. Consistent with this idea, microglia have been shown to associate preferably with shrinking spines after changes in sensory experience *in vivo*^16^.

In conclusion, we provide clear evidence that microglia increase their scanning activity and modify their interactions with dendritic spines during hippocampal LTP. While this altered behavior may implicate microglia in synaptic remodeling in the healthy adult brain, future studies need to clarify whether microglial processes are targeting a specific subset of synapses and what the functional consequences for these synapses might be.

## Materials and Methods

### Animals

Cx3Cr1^+/eGFP^-Thy1^+/eYFP^ mice were used, where GFP is expressed in all microglia under the fractalkine receptor (Cx3Cr1) promoter^21^ and YFP in a subpopulation of principal neurons in the hippocampus under the Thy1 promoter^22^ (Jackson Labs, Bar Harbor, ME). All experiments were carried out in accordance with the Directive 2010/63/EU of the European Parliament and approved by the Ethical Committee of Bordeaux (#50120201).

### Acute slice preparation

Acutely prepared hippocampal slices were obtained from 28–40 days old mice of both sexes. Mice were anesthetized with isoflurane prior to decapitation. Brains were quickly removed and placed in ice-cold, oxygenated (95% O_2_ and 5% CO_2_) sucrose-based artificial cerebrospinal fluid (ACSF) containing (in mM): 210 sucrose, 10 glucose, 2 KCl, 26 NaHCO_3_, 1.25 NaH_2_PO_4_, 0.1 CaCl_2_ and 6 MgCl_2_ (pH 7.4, osmolarity ~315 mOsm/L). Sagittal slices were cut (350 μm thick) and incubated for 1 hour at 33 °C in carbogenated (95% O_2_, 5% CO_2_) ACSF containing (in mM): 124 NaCl, 3 KCl, 26 NaHCO_3_, 1.25 NaH_2_PO_4_, 10 glucose, 2 CaCl_2_ and 1 MgCl_2_ (pH 7.4, osmolarity ~305 mOsm/L). Subsequently, slices were stored at room temperature (RT) and used until 4 hours after preparation. Experiments were performed in a submerged recording chamber at 33 °C with continuous perfusion (3–4 ml/min) of carbogenated ACSF.

### Slice electrophysiology

Schaffer collateral fibers were electrically stimulated at 0.05 Hz and evoked field excitatory postsynaptic potentials (fEPSP) were recorded in the *Stratum radiatum* of hippocampal CA1. Recording electrodes were carefully positioned in the slice and placed at depths where imaging was performed (Fig. 1A). NMDAR-dependent LTP was induced by applying a high-frequency stimulation (HFS) protocol consisting of 1 s trains of 100 Hz stimulation repeated two times 20 s apart. For APV experiments, 50 μM APV (Tocris Biosciences), an NMDAR antagonist, was continuously bath applied.

### Two-photon imaging

Microglia and neuronal dendrites were imaged in the *Stratum radiatum* of CA1 using a commercial two-photon microscope (Prairie Technologies). For simultaneous two-color imaging of GFP (microglia) and YFP (neurons) the two-photon laser (Ti:sapphire, Mai Tai, Spectra Physics) was tuned to 900 nm. Images were acquired using a 40 × 1.0 NA water immersion objective (Plan-Apochromat, Zeiss). Laser power ranged from 10–25 mW in the focal plane. The fluorescence signal was spectrally divided into two channels by a dichroic mirror at 514 nm and collected in a non-descanned way by PMT detectors. Z-stacks of 10 μm with 1 μm step size were collected consecutively every 30 s for 80 min. Imaging was performed at least 50 μm below the slice surface, where microglia are less likely to be affected by the slicing procedure. Images were acquired at 512 × 512 pixels with a pixel size of 200 nm, a dwell-time of 3.6 μs and two running frame averages.

### Image processing and analysis

Images were processed using MATLAB and ImageJ. Spectral unmixing of the fluorophores, GFP and YFP, was carried out using a custom-written MATLAB routine (courtesy of F. Nadrigny^23^). Time-lapse image series based on concatenated single maximum intensity z-projections (MIP) were prepared independently for both fluorophores. To correct for x-y drifts during time-lapse image acquisition, the ImageJ plug-in MultiStackReg was applied. For further analysis spectrally unmixed and drift-corrected GFP and YFP channels were merged. For illustration purposes brightness and contrast of the images were adjusted and a median filter was applied.

To assess global changes in microglial morphological dynamics we defined two parameters, the microglial scanning density and the microglial domain (Fig. 1D,G). The time-lapse images were binned into time windows of 20 min, resulting in one baseline (−20–0 min) and three post HFS bins (0–20, 20–40, 40–60 min). Control experiments were carried out similarly without HFS. The resulting cumulative MIPs over 20 min, based on 40 time points, served as a measure of microglial morphological dynamics. Thus, every GFP-positive pixel represents a position in space, which was visited by a microglial process during the respective time window. For evaluation of the scanning density a line was manually drawn around the microglia. To measure the microglial domain the most distant process tips were linked. The changes in each parameter over time were expressed in percent relative to the baseline values.

Microglial process velocity was assessed by tracking single microglial process tips in consecutive MIPs using the ImageJ plugin mTrackJ^24^. Changes were determined by comparing the average velocity of 10 motile microglial processes before and after HFS (i.e. in 20 min baseline and 40–60 min after HFS). As motile processes underlie a high turnover we were not able to analyze the same process in the two conditions. Processes at the edge of the z-stack were not included in the analysis.

Changes in the number of microglial processes were evaluated by comparing MIPs chosen at one time point during the baseline (0′–20′) and after HFS (40′–60′). To minimize variations due to shifts in the z-plane, the two images were chosen in order to have similar cell body shape and major 1^st^ order branches. The spectrally-unmixed image was first binarized applying a custom-written ImageJ plugin, which is based on wavelet-thresholding, and then skeletonized. The number of microglial processes was then extracted using the ImageJ plugin AnalyzeSkeleton^25^.

A contact between a microglial process and a dendritic spine was defined as a physical apposition closer than 2 pixels (i.e. ≤400 nm) in the same z-plane. The number of microglia-spine contacts in each time window was normalized to 100 μm dendrite and the contact durations were evaluated by number of frames and subsequently translated in minutes. Changes were assessed comparing measurements obtained in periods before and after LTP induction.

### Statistics

To assess statistical differences in the electrophysiological data Students t-tests were used. One-way Anova with repeated measures and multiple comparisons with Turkey post-hoc test were applied to test changes in microglial morphological dynamics, taking into account the factor of time. Paired t-tests were applied to test changes in microglial process velocity and microglia-spine interactions. Wilcoxon paired test was applied on the non-Gaussian distributed number of microglial processes. The number of slices and microglial cells analyzed is indicated in the text and the respective figures. Data are represented as mean ± sem. The number of microglial processes was presented by median, 25^th^ and 75^th^ percentile. The percentage of spines contacted by a microglial process was represented by mean ± SD to indicate the variability. The significance level was chosen to be 0.05. The asterisks in the figures denote *p < 0.05, **p < 0.01 and ***p < 0.001.

## Additional Information

**How to cite this article**: Pfeiffer, T. *et al*. Induction of hippocampal long-term potentiation increases the morphological dynamics of microglial processes and prolongs their contacts with dendritic spines. *Sci. Rep.* **6**, 32422; doi: 10.1038/srep32422 (2016).

## Supplementary Material

Supplementary Information
