# Supplementary material for: Induction of hippocampal long-term potentiation increases the morphological dynamics of microglial processes and prolongs their contacts with dendritic spines
Source: Sci Rep. 2016 Sep 8;6:32422. doi: 10.1038/srep32422 (PMC5015055; doi:10.1038/srep32422)
Supplement: Supplementary Information [file srep32422-s1.doc]

**Supplementary information**

**Induction of hippocampal long-term potentiation increases the morphological dynamics of microglial processes and prolongs their contacts with dendritic spines**

Thomas Pfeiffer^1,2^, Elena Avignone^1,2^* and U. Valentin Nägerl^1,2^*^+^

^1^Interdisciplinary Institute for Neuroscience, UMR 5297 CNRS, Bordeaux, France

^2^Université de Bordeaux, Bordeaux, France

*Co-senior author

^+^ Corresponding author


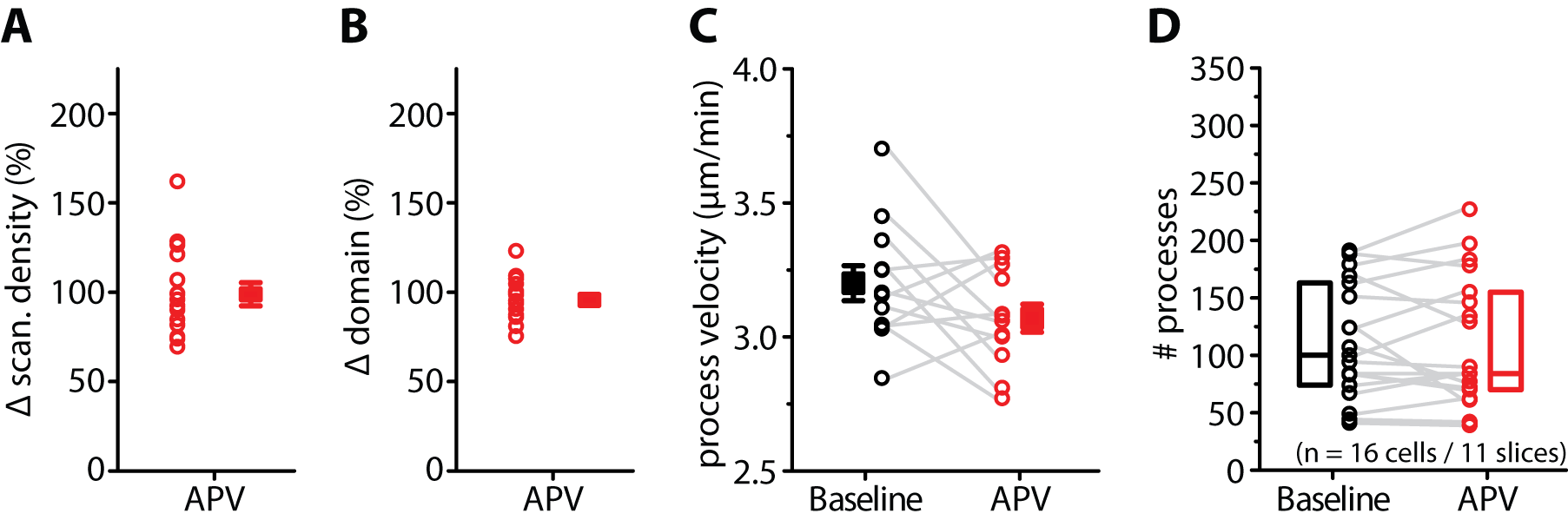


**Supplementary Figure S1.** **Microglial morphological dynamics are not changed in the presence of the NMDA receptor antagonist APV.**

***A, B,*** The scanning density of microglia (A) and the size of its domain (B) remained unaffected by the application of 50 µM APV. For this, 20 min of drug-free baseline were compared to 20 min in the presence of APV (scanning density: 99.2 % ± 6.17 %, p = 0.45, mean ± sem, paired t-test, compared to baseline, n = 16 cells / 11 slices; domain: 95.74 % ± 3.07 %, p = 0.08, mean ± sem, paired t-test, compared to baseline). ***C, D,*** Similarly, microglial process velocity (C) (baseline: 3.2 ± 0.07 µm/min; APV: 3.07 ± 0.05 µm/min; p = 0.11, mean ± sem, paired t-test , n = 11 slices) and the number of processes per microglia (D) (baseline: median 100.0 [74, 163]; APV: median 84 [70, 155]; median [25^th^ and 75^th^ percentile], Wilcoxon paired test, p = 0.79, n = 11 slices) were not affected by the application of APV.
